# Neurofibromatosis Type 2 Presenting as Symptomatic Gallbladder Hydrops: A Rare Case Report and Literature Review

**DOI:** 10.1155/crpe/7680840

**Published:** 2024-11-20

**Authors:** Bassel Hafez, Joudie Sahar Alwan, Walid El Hout, Karim Koussa, Tamara El Annan, Dolly Noun, Ahmad Zaghal

**Affiliations:** ^1^Department of Surgery, American University of Beirut Medical Center, Beirut, Lebanon; ^2^Department of Emergency Medicine, American University of Beirut Medical Center, Beirut, Lebanon; ^3^The Christie NHS Foundation Trust, Manchester, UK; ^4^Department of Diagnostic Radiology, American University of Beirut Medical Center, Beirut, Lebanon; ^5^Department of Pediatrics and Adolescent Medicine, American University of Beirut Medical Center, Beirut, Lebanon

**Keywords:** cholecystectomy, gallbladder hydrops, gallbladder schwannoma, neurofibromatosis type 2, schwannoma

## Abstract

Neurofibromatosis type 2 (NF2), also known as NF2-related schwannomatosis (SWN), is a rare dominantly inherited genetic disorder mainly characterized by the presence of vestibular schwannomas (VSs) in addition to a range of other tumors that affect both the central and peripheral nervous systems. These tumors include cranial, spinal, peripheral nerve, and intradermal schwannomas, cranial and spinal meningiomas, and intrinsic central nervous system (CNS) tumors, usually spinal ependymomas. Juvenile cataracts are also common in patients with NF2, with most symptoms at presentation being hearing loss and visual disturbances. We present the case of a previously healthy 12-year-old girl who presented with postprandial right upper quadrant pain and was found to have a large hydrops of the gallbladder on ultrasound scan of the abdomen. Pathology of the gallbladder post laparoscopic cholecystectomy showed diffuse involvement of the gallbladder by a benign nerve sheath tumor that was suggestive of schwannoma. Further testing confirmed the diagnosis of NF2. This case helps shed light on unusual NF2 symptoms and underscores the importance of recognizing atypical presentations for timely intervention and management. It also adds value to a multidisciplinary approach in diagnosing and managing NF2.

## 1. Introduction

Type 2 neurofibromatosis is an autosomal dominant disorder affecting the nervous system causing multiple tumor growth called schwannomas and meningiomas [1]. It is caused by mutations in the NF2 tumor suppressor gene on chromosome 22. The incidence of NF2 is about 1 in 25,000 to 40,000 individuals [2]. Schwannomas are encapsulated benign tumors that grow from peripheral nerves and are made of benign neoplastic Schwann cells [3]. When occurring sporadically, they tend to grow as solitary tumors and can affect people of all ages with no predilection to sex or race [4]. However, when numerous schwannomas are discovered, they tend to be associated with NF2. Whether sporadic or inherited, these tumors affect the peripheral nervous system and are preferentially found on the head, neck, and flexor surfaces of the extremities [3]. Uncommon locations of schwannoma growth have been described in the literature and include retroperitoneum, perianal, or even vagina [5–7]. Herein, we present an intriguing case involving an atypical presentation of a schwannoma—specifically, a schwannoma in the gallbladder in a pediatric patient—which resulted in the incidental discovery of NF2 disease.

## 2. Case Report

This is a case of a previously healthy 12-year-old girl who presented to medical attention for recurrent episodes of biliary colic of two years duration. The patient described her pain as dull, moderate to severe in intensity, located in the right upper abdominal quadrant radiating to her interscapular region. The pain is exacerbated by fatty meals; the patient denied any relieving factors. The patient's past medical, past surgical, and family history was negative.

Physical examination showed a soft, nondistended and nontender abdomen, mild fullness in the right upper quadrant, and negative Murphy's sign. No signs of obstructive jaundice were noted.

Laboratory values, including liver function tests, were all within normal range. There was no evidence of cholestasis (Table 1).

**Figure 1:**
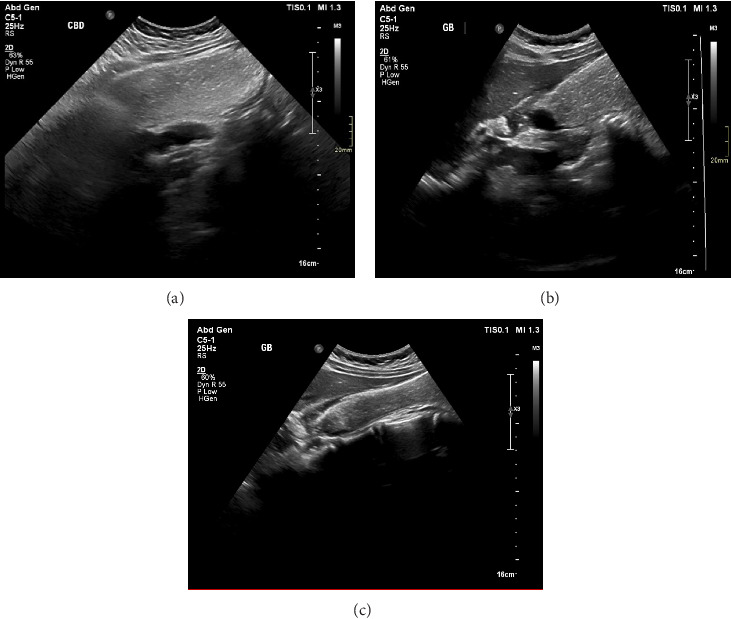
Grayscale ultrasound images of the gall bladder revealing severe gallbladder hydrops and distension reaching 18.5 × 6.1 in dimensions filled with sludge and calculi (a) and apparent concentric wall thickening measuring 6 mm involving the gallbladder neck (b, c).

Ultrasound of the gallbladder showed a large gallbladder hydrops reaching 18.5 × 6.1 cm, filled with sludge and calculi, and evident concentric wall thickening with a diameter measuring 6 mm involving the gallbladder neck (Figure 1), with no signs of either intra- or extra-hepatic biliary duct dilation. Differential diagnoses, including cholecystitis, choledocholithiasis, hepatitis, pancreatitis, hemolysis, hyperlipidemia, biliary obstruction, gallbladder cancer, porcelain gallbladder, acalculous gallbladder disease, and chronic liver disease, have been ruled out based on blood work and ultrasonographic findings.

**Figure 2:**
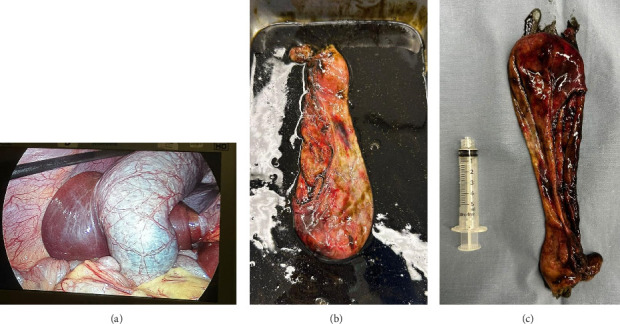
Intraoperative specimens. (a) Intraoperative laparoscopic view of gall bladder hydrops when retracted cephalad. (b) Resected gall bladder revealing sludge in basin. (c) Resected gall bladder when compared to a 5-cc syringe.

The decision to proceed with surgery was made, and the operation occurred on the second day following the diagnosis. Laparoscopic cholecystectomy was performed using a 10 mm umbilical trocar, a 5 mm epigastric trocar, and two 5 mm right upper quadrant trocars. The hydrops was deflated using a long laparoscopic needle introduced through the fundus of the gall bladder. After identifying the triangle of safety, the cystic artery and duct were clipped, and the gallbladder was shaved off the liver bed using the laparoscopic hook with electrocautery. The gallbladder was exteriorized via the umbilical incision after a minimal extension of the fascial opening of the umbilical trocar site. The gall bladder was retrieved intact (Figure 2). Operative time was around 2 h. The patient had an uneventful postoperative course and was discharged home one day later.

The gross macroscopic examination of the surgical specimen showed a gallbladder measuring 17 × 6 × 2 cm with a congested serosa and an average wall thickness of 0.4 cm. Multiple firm sessile polypoid protrusions were observed within the gallbladder, ranging irregularly in shape from 0.5 cm to 3.5 cm. These polyps were more numerous and larger in the neck of the gallbladder. Microscopic analysis of the specimen revealed a diffuse spindle cell proliferation primarily centered in the mucosa, extending into the muscular wall and slightly into the subserosa. The spindle cell proliferation exhibited moderate cellularity with subtle nuclear palisading in few areas. The cells featured tapered nuclei, abundant wavy eosinophilic cytoplasm, and indistinct cell borders, with scattered nuclear atypia. No mitotic activity or necrosis was identified, and there was no evidence of acute cholecystitis or malignancy. Further histopathologic examination confirmed diffuse involvement of the gallbladder by a benign nerve sheath tumor strongly positive for S100, indicative of a schwannoma. Subsequently, the patient was scheduled for follow-up and referred to pediatric oncology, pediatric ophthalmology, and pediatric neurology services.

The ophthalmology assessment revealed epiretinal membrane (ERM) in both eyes, a feature typically found in NF2 patients, along with anisometropia, and amblyopia in the right eye, a macular scar in the right eye, and a chalazion in the left upper eyelid. There was no evidence of retinal hamartoma nor RPE. Since vision was stable, there was no indication to operate on the ERM. The ophthalmological evaluation raised suspicion for a possible NF2 diagnosis; however, before affirming this suspicion, toxoplasma IgM/IgG tests were ordered to rule out toxoplasma infection, yielding negative results. Appropriate treatment was given to the patient with lid hygiene with eyelid wipes and warm compresses administered four times daily.

**Figure 3:**
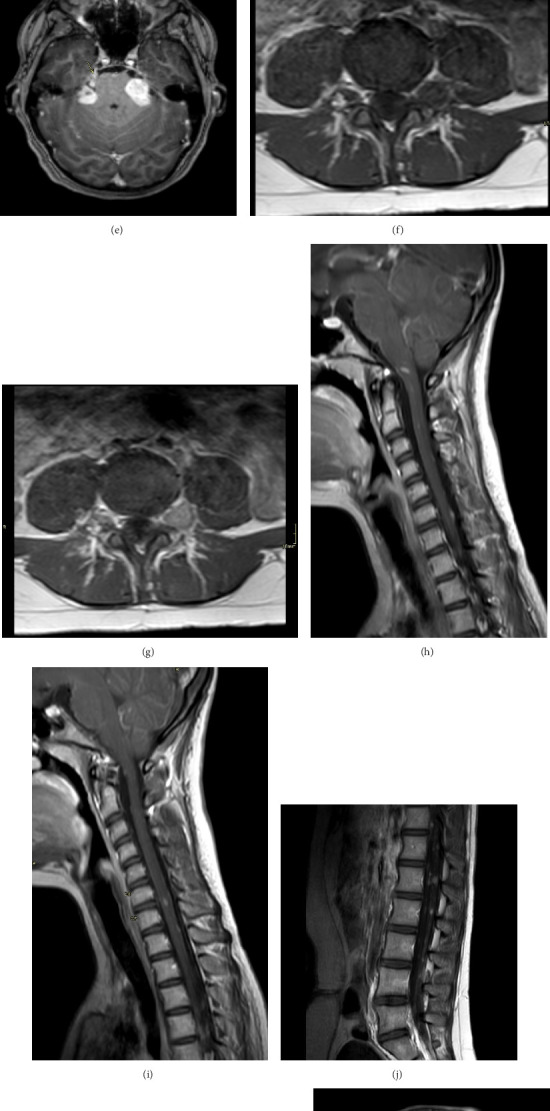
MRI images revealing multiple schwannomas. (a–c) Axial FLAIR, axial T2∗, and axial T1W images post gadolinium administration at the level of the cerebellopontine angles: bilateral solid soft tissue masses in the cerebellopontine angles, showing intracanalicular component with widening the porus acusticus (trumpeted IAM sign) and causing mass effect on the lateral aspect of the pons, middle cerebellar peduncles and cerebellum, with narrowing of the fourth ventricle. Both lesions show high FLAIR signal, intense enhancement post contrast with no diffusion restriction (not shown), and contain foci of hemorrhage, consistent with bilateral vestibular schwannomas. (d, e) Axial T1W image post gadolinium administration shows small enhancing schwannomas along the trigeminal nerve, bilaterally (right is shown), inferior to the Meckel's cave, measuring approximately 8 mm. (f, g) Axial T1W images pre and post gadolinium administration at the L4-L5 level show a 1.3 × 1.4 cm enhancing lesion on the left side in keeping with extraforaminal neurofibroma or schwannoma. (h–j) T1W images of the spine post gadolinium administration show multiple enhancing lesions at the level of the craniocervical junction and within the spine, some of which are intramedullary (at C2-C3, C6-C7) and others along the surface of the cord, corresponding to ependymomas and neurofibromas. Note is made of mild cerebellar tonsillar herniation. (k) Axial T1W image post gadolinium administration at the level of L2 showing 6 mm enhancing lesion along the nerve roots. (l) Axial T1W images post gadolinium administration: subcutaneous enhancing lesions at the level of the left frontal and parietal scalp, representing subcutaneous neurofibromas. (m, n) Axial T1W images post gadolinium administration showing a 7 mm extra-axial, broad-based, enhancing lesion arising from the right anterior clinoid process and another 6 mm lesion along the falx anteriorly in keeping with meningiomas.

**Figure 4:**
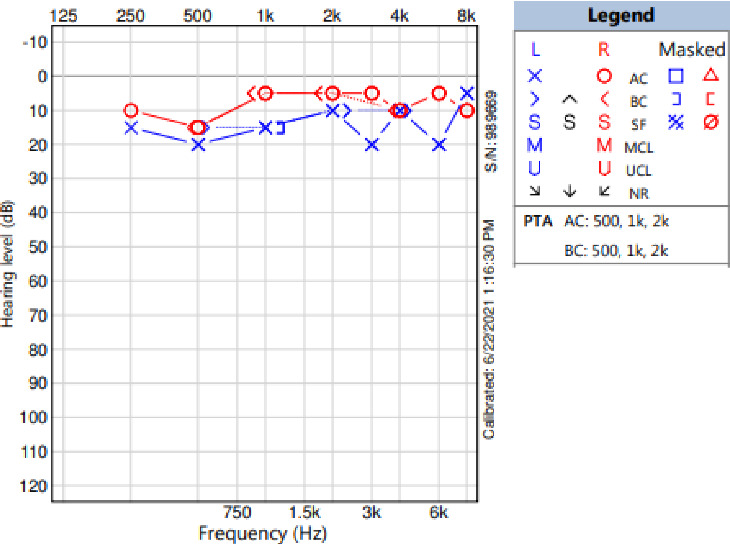
Audiometry.

During the neurological evaluation, her detailed physical examination revealed full motor powers with subtle multiple neurofibromas over the scalp and one over left foot. The patient's atypical presentation raised further suspicions of NF2 due to her young age and involvement of the gallbladder, prompting a solo whole genome sequencing test to be ordered to rule out associations with NF2. The test confirmed the diagnosis of NF2 by identifying an NF2 variant c.703G > T p. (Gly235∗) which creates a premature stop codon. It is classified as likely pathogenic (class 2) according to the recommendations of the genetic test called CENTOGENE and the American College of Medical Genetics and Genomics (ACMG). Consequently, following diagnosis, MRIs of the brain and spine were performed and revealed bilateral vestibular and trigeminal nerve schwannomas, right anterior clinoid meningioma, ependymomas and neurofibromas at the craniocervical junction and at several levels along the spine both intramedullary and along the surface of the spine, and extraforaminal schwannomas at several levels of the lumbar spine in addition to subcutaneous neurofibromas of the left frontal scalp (Figure 3), along with a referral to neurosurgery. The neurosurgery team determined that surgical intervention was unnecessary at this stage but recommended a baseline hearing assessment. Warble tone audiometry revealed normal hearing sensitivity at all tested frequencies with excellent word recognition score (WRS) bilaterally. A positive rollover was noted on the left side. Audiometry showed asymmetry of 10–20 dB was noted with poorer thresholds in the left ear (Figure 4).

Oncology presented the case at the tumor board meeting, and bevacizumab was suggested as an option for management of the VS. After thorough discussion and review of the possible side effects associated with treatment, and in the setting of minimal hearing loss, the family and the treating physician opted to monitor very closely and consider bevacizumab in case of deterioration, with plans to re-evaluate in three months.

In the meantime, the patient was referred to plastic surgery for the resection of forehead and scalp neurofibromas.

At the three-month follow-up, MRI findings revealed a 3 mm increase in vestibular masses bilaterally, but the ophthalmological evaluation and audiogram remained unchanged, and the patient remained asymptomatic. During the multidisciplinary meeting, the neurosurgery and oncology teams recommended initiation of bevacizumab; however, the parents decided to continue with observation since hearing was still preserved and the patient was still asymptomatic. The oncology team decided to go for close follow-up with careful reevaluation in 2-3 months.

At the subsequent follow-up, the patient showed signs of progression and worsening of hearing loss. Thus, the decision to start bevacizumab was agreed during a multidisciplinary meeting and regular follow-up appointments were scheduled every 3 months, including serial MRI studies, to monitor progression and response to treatment.

## 3. Discussion

NF2 is an inherited genetic disorder that is characterized by VSs as its main presentation. MISME (Multiple Inherited Schwannomas, Meningiomas, and Ependymomas), an older yet expressive term, refers to the occurrence of multiple schwannomas, meningiomas, and ependymomas, highlighting the presence of various tumor types along the CNS. However, schwannomas arising from the gall bladder are extremely rare and have never previously been reported in patients with NF2. Solitary schwannomas of the gallbladder nonetheless have been reported, with only 6 cases discussed to date [8–13] (Table 2).

Patient presentations varied from asymptomatic patients to patients with jaundice, or biliary colic. With an average age of onset of 18–24 years, almost all patients develop bilateral vestibular schwannomas (BVSs) by age 30. NF2 is considered an adult-onset disease and is not as well recognized as NF1 in this population. Café-au-lait occur only on 1%-2% of patients. Childhood presentation has been described and may be associated with a more severe course, probably reflecting the underlying genetic mutation [14].

Symptoms at diagnosis usually result from CNS manifestations or ocular or skin findings [15–17].

It is important to distinguish SWN and NF2 which share a significant phenotypic overlap but differ in terms of genetic causes. Genetic culprits of the former include SMARCB1 and LZTR1 also falling within the 22q region and the NF2 gene [18].

SWN is characterized by the presence of more than 2 schwannomas and the absence of VSs. Updated guidelines were published recently [19].

NF2 is associated with significant rate of mosaicism, thus explaining the absence of family history in many patients and failure of detecting the genetic mutation in many others.

The detection of a peripheral schwannoma at a very unusual location and a very young age in our patient prompted, justifiably, further investigation, based on which the Manchester diagnostic criteria were fulfilled, thus establishing the diagnosis of NF2. The genetic test further ascertained the diagnosis.

Several imaging modalities are used to achieve preoperative diagnosis. An unenhanced CT scan depicts a schwannoma as a well-defined hypoattenuating area, and CECT shows peripheral enhancement with an irregular pattern. Delayed peripheral enhancement until the late venous phase on the CT scan reflects a fibrous capsule and an internal fibrillary element [9, 20, 21]. The ultrasound features of this lesion are nonspecific and have been reported differently in previous cases. Ohta et al. reported high echoic lesions with gallbladder thickening at the fundus with a diagnosis of cholecystolithiasis made preoperatively [8]. Liu et al. reported a well-defined round isoechoic mass originating from the gallbladder wall on grayscale US, and no intralesional blood flow signals were visible on color Doppler US [9]. Although the lesion was larger than 2 cm in diameter, the wall beneath the mass was intact. In our case, ultrasound of the gallbladder was performed and showed severe gallbladder hydrops filled with sludge and calculi and evident concentric wall thickening involving the gallbladder neck. We believe that the diffuse involvement of the gall bladder with benign nerve sheath tumors contributed to the gall bladder hydrops and caused the patient's symptoms.

Based on the clinical presentation and the case, our patient would be classified as having a group 3 phenotype according to Halliday, Emmanouil, and Evans and the English NF2 research group, associated with greater tumor burden and poorer outcomes, and is thus most likely expected to require intervention and treatment with bevacizumab or other targeted therapy [22].

Recent advances in molecular characterizations led to the identification of targeted therapies such as bevacizumab and lapatinib which can positively alter quality of life of these patients, the care of whom requires a multidisciplinary approach and appropriate genetic counseling [23].

Efforts to understand the molecular pathways driving tumor development in NF2 have led to the reconsideration of existing cancer drugs for potential use in NF2 patients. Five treatments have been proposed based on preclinical data, including bevacizumab, lapatinib, erlotinib, and everolimus. Bevacizumab, in particular, has been associated with positive outcomes in retrospective studies, demonstrating hearing improvement and tumor shrinkage in over 50% of progressive VSs. In a study involving 16 patients, bevacizumab was linked to a median time to tumor progression increase from 5.6 months before treatment to over 29.3 months. However, the need for intravenous injections and the potential for long-term adverse events such as hypertension, proteinuria, and hemorrhage are notable drawbacks [24]. Lapatinib showed promise in a Phase II trial, while erlotinib did not demonstrate significant radiographic or hearing responses. Everolimus, though not associated with tumor shrinkage, has shown potential for increasing time to tumor progression in selected cases [25]. Currently, bevacizumab stands as the only drug recommended for selected NF2 patients [26].

A recent clinical study investigated the use of vascular endothelial growth factor receptor (VEGFR) peptide vaccine in seven patients with progressive NF2-derived schwannomas. The study found that the vaccine induced both VEGFR1-specific and VEGFR2-specific cytotoxic T-lymphocytes (CTLs) in six patients. Results showed improvements in hearing in 2/5 patients, with increases in WRSs, as well as tumor volume reductions of ≥ 20% in two patients, one of whom had not responded to bevacizumab. Additionally, no severe adverse events related to the vaccine were reported, and significant reductions in VEGFR expression in schwannomas were observed. The study demonstrated the safety and preliminary efficacy of VEGFR peptide vaccination in patients with NF2, suggesting potential benefits for this immunotherapy approach [27].

In a prospective multicenter Phase II study, maintenance therapy with bevacizumab (5 mg/kg every 3 weeks) showed promising efficacy in preserving hearing and stabilizing tumor growth over 18 months in individuals with NF2-related SWN and hearing loss due to VSs. The treatment demonstrated high rates of freedom from hearing loss and tumor growth, along with stable NF2-related quality of life, and was generally well tolerated with minimal adverse events leading to treatment discontinuation [28].

In our review of SWN and NF2, it is essential to address the challenges in diagnosing rare tumors. A case by Shen et al. involving a 21-year-old pregnant woman with a gallbladder mass initially suggested malignancy based on imaging and elevated tumor markers, leading to an urgent abortion and cholecystectomy. However, the mass was found to be a benign schwannoma postoperatively [29]. This underscores the importance of accurate differential diagnosis in evaluating gallbladder masses in the pediatric and adult population to prevent unnecessary invasive procedures and ensure appropriate patient management.

To our knowledge, this is the first case report of a gallbladder schwannoma unveiling the diagnosis of NF2 in a child. All other previous reports discussed solitary schwannomas of the gallbladder, but none were concurrent with other tumors of the CNS suggestive of NF2 upon further follow-up.

## 4. Conclusion

In summary, NF2-related SWN is a complex genetic disorder characterized by the presence of various tumors affecting the central and peripheral nervous systems. This case report highlights the atypical presentation of symptomatic gallbladder hydrops in a 12-year-old girl, leading to the diagnosis of NF2 after the identification of a benign nerve sheath tumor involving the gallbladder. To date, bevacizumab appears to be the most effective treatment option. This emphasizes the need for clinicians to maintain a broad differential diagnosis for unusual presentations and to explore potential associated conditions through thorough testing when possible. Early recognition is also crucial, as it may prevent severe consequences such as hearing loss and visual impairments. Additionally, this case underscores the value of a multidisciplinary approach in managing complex conditions, ensuring tailored and comprehensive care for optimal patient outcomes.

## Figures and Tables

**Table 1:** Laboratory values of the patient.

	Latest reference range and units	Labs on admission
Bilirubin total	0.0–1.2 mg/dL	0.6
Bilirubin direct	0.0–0.3 mg/dL	0.2
Alkaline phosphatase	20–385 IU/L	140
SGPT (ALT)	0–50 IU/L	21
SGOT (AST)	0–50 IU/L	24
Gamma-glutamyl transferase (GGT)	10–50 IU/L	14
Lipase	13–60 U/L	18

**Table 2:** Different presentations of gall bladder schwannomas.

Reference	Year	Symptom	Imaging	Location within gallbladder	Preoperative diagnosis	Treatment	Sex	Age	NF2 association
Yamagiwa [10]	1991	Jaundice	NA	Neck	Bile duct cancer	Cholecystectomy	M	58	NA
Matsuoka et al. [11]	1996	Asymptomatic	US, MRI, CT, and endoscopy	Fundus	Adenomyomatosis	Cholecystectomy + hepatectomy	M	74	NA
Ren, Wu, and Hao [12]	2001	Vague pain	US and CECT	Neck	Tumor of the gallbladder	Cholecystectomy	F	26	NA
Čolović et al. [13]	2003	NA	NA	Whole	Tumor of the gallbladder	Cholecystectomy	F	61	NA
Ohta et al. [8]	2008	Asymptomatic	CT and US	Fundus	Cholecystolithiasis	Cholecystectomy	M	58	No association
Liu et al. [9]	2012	Asymptomatic	CEUS and CECT	Neck	Adenomyomatosis	Cholecystectomy	M	55	No association
Lin et al. [30]	2018	Epigastric pain and jaundice	MRI	Whole	Laparoscopic CBD exploration and cholecystectomy	Schwannomas of the gallbladder, cholecystolithiasis, and choledocholithiasis	M	70	NA
Shen et al. [29]	2023	Upper abdominal pain	US, CT, MRI, and PET-CT	Body	Malignant tumor of the gallbladder with liver invasion	Cholecystectomy + abortion of pregnancy	F	21	NA
Current case	2023	Biliary colic	US gallbladder	Neck	Gallbladder hydrops and cholecystitis	Cholecystectomy	F	12	Confirmed NF2 diagnosis

## Data Availability

No supporting data files are available. All data supporting the findings of this study are included within the article.
